# Stress impacts sensory variability through cortical sensory activity motifs

**DOI:** 10.1038/s41398-020-0713-1

**Published:** 2020-01-21

**Authors:** Alexander McGirr, Jeffrey LeDue, Allen W. Chan, James D. Boyd, Paul D. Metzak, Timothy H. Murphy

**Affiliations:** 1 Department of Psychiatry, University of Calgary, Calgary, AB Canada; 2 Hotchkiss Brain Institute & The Mathison Centre for Mental Health Research and Education, University of Calgary, Calgary, AB Canada; 3 Department of Psychiatry, University of British Columbia, Vancouver, BC Canada; 4 Djavad Mowafaghian Brain Research Centre, University of British Columbia, Vancouver, BC Canada; 5 Department of Psychiatry, University of Alberta, Edmonton, AB Canada

**Keywords:** Neuroscience, Depression

## Abstract

Medically unexplained symptoms in depression are common. These individual-specific complaints are often considered an ‘idiom of distress’, yet animal studies suggest that cortical sensory representations are flexible and influenced by spontaneous cortical activity. We hypothesized that stress would reveal activity dynamics in somatosensory cortex resulting in greater sensory-evoked response variability. Using millisecond resolution in vivo voltage sensitive dye (VSD) imaging in mouse neocortex, we characterized spontaneous regional depolarizations within limb and barrel regions of somatosensory cortex, or spontaneous sensory motifs, and their influence on sensory variability. Stress revealed an idiosyncratic increase in spontaneous sensory motifs that is normalized by selective serotonin reuptake inhibitor treatment. Spontaneous motif frequency is associated with increased variability in sensory-evoked responses, and we optogenetically demonstrate that regional depolarization in somatosensory cortex increases sensory-evoked variability for seconds. This reveals a putative circuit level target for changes in sensory processing and for unexplained physical complaints in stress-related psychopathology.

## Introduction

Depressive disorders are associated with somatic symptoms for which medical causes cannot be identified^1^. Even brief stresses result in new onset somatic symptoms^2^. These symptoms are idiosyncratic and fluctuant over time, both in terms of anatomical location and quality^3^. Sensory testing in individuals with major depression reveals both increases and decreases in sensory thresholds^4^, suggesting an alteration of sensory processing. Yet, the neurobiology underlying altered sensory experience in depression and stress-related pathology remains unclear, and accordingly it is often considered an ‘idiom of distress’.

Imaging and electrophysiology in model species suggests that cortical sensory representations are flexible, as identical stimuli do not reproducibly elicit the same responses^5–8^. In part, this is related to a bidirectional relationship between spontaneous activity in cortex and sensory stimuli. Sensory events can silence spontaneous activity dynamics for seconds^5^, yet there is also evidence that cortical state influences the reliability of sensory-evoked responses. When spontaneous regionally synchronized depolarizations occur in sensory cortex, the magnitude of sensory-evoked responses are decreased compared to the same sensory stimulus delivered while the region is hyperpolarized with minimal membrane potential variance^5,7^. Accordingly, trial-to-trial variability is increased when sensory stimuli are delivered surrounding a depolarized state, whereas they are more reliable during a hyperpolarized state^9^.

There is a large body of literature to support altered resting state activity in human depression and excessive activity in ruminative ‘egocentric’^10,11^ and interoceptive^12^ brain networks^13,14^. We hypothesized that resting state activity alterations in depressed humans, and their murine homologue in spontaneous activity^15^, would reveal activity dynamics sensitive to stress in sensory regions of the cortex to inform unexplained somatic symptoms. However, the individual-specific nature of the human phenomenon of interest poses an additional challenge to animal modelling, as in the absence of objectively verifiable pathology the location of somatic complaints cannot be localized a priori. To resolve this challenge, we built on in vivo imaging^7,10^ and electrophysiological^11,16,17^ studies in rodent sensory cortex identifying propagating waves of depolarization that respect reproducible spatiotemporal features. These activity dynamics have been termed spontaneous motifs^11,18^. When the field of view is expanded to include a large area of neocortex, in vivo imaging reveals spontaneous motifs originating from diverse functional regions of cortex, and those originating within primary somatosensory areas have a distinctive evolution resembling sensory experience^10,18,19^. Thus, by capturing spontaneous activity from a wide expanse of dorsal neocortex, we can identify diverse spontaneous sensory motifs. We hypothesized that these would not be uniformly affected by stress, but that as in depressed humans, at least one sensory motif would be idiosyncratically stress susceptible and increase in frequency.

Here, we show that spontaneous cortical sensory motifs are susceptible to stress, that this circuit level susceptibility is idiosyncratic and related to the behavioural sequelae of stress. These sensory motifs, in turn, impact the variability of sensory-evoked responses in cortex.

## Methods and materials

### Experimental strategy

We aimed to characterize spontaneous cortical dynamics and sensory-evoked variability. We acquired spontaneous brain activity with 6.67 ms (150 Hz) temporal resolution for a total of 50,005 frames (repeatedly if we also imaged under quiet wakefulness), and performed standardized electrical or piezo sensory stimulation protocols to obtain individualized templates of sensory experience.

Animals were pseudorandomly assigned to experimental conditions. Behavioural experiments and imaging acquisitions as well as processing were performed blinded to experimental condition. Analyses were not blinded to experimental condition.

### Animals

We utilized C57BL/6J and CD1 mice from Charles River in chronic social defeat (CSD) experiments. C57BL/6J mice for maternal deprivation (MD) experiments were bred in our facility. We also utilized the Ai85 transgenic mice (EMX-CaMKII-iGluSnFR) for simultaneous imaging and optical stimulation. These animals express a recombinant sensor based on a non-functional extracellular glutamate receptor. EMX-CaMKII-iGluSnFR transgenic mice^20^ expressing iGluSnFR in excitatory cortical neurons were generated by crossing homozygous B6.129S2- Emx1^tm1(cre)Krj^/J strain (Jax #005628) and B6.Cg-Tg(CamK2a-tTA)1Mmay/DboJ strain (Jax #007004) with hemizygous B6; 129S-Igs7^tm85(teto-iGluSnFR)Hze^/J strain (Jax #026260)^20,21^.

The housing facility had a 12:12 light cycle. Mice were housed in groups of three to five. All mice had ad libidum access to water and standard laboratory mouse diet. The animal protocols were approved by the University of British Columbia Animal Care Committee and were in accordance with guidelines set forth by the Canadian Council for Animal Care.

### Voltage sensitive dye surgery

**Fig. 1:**
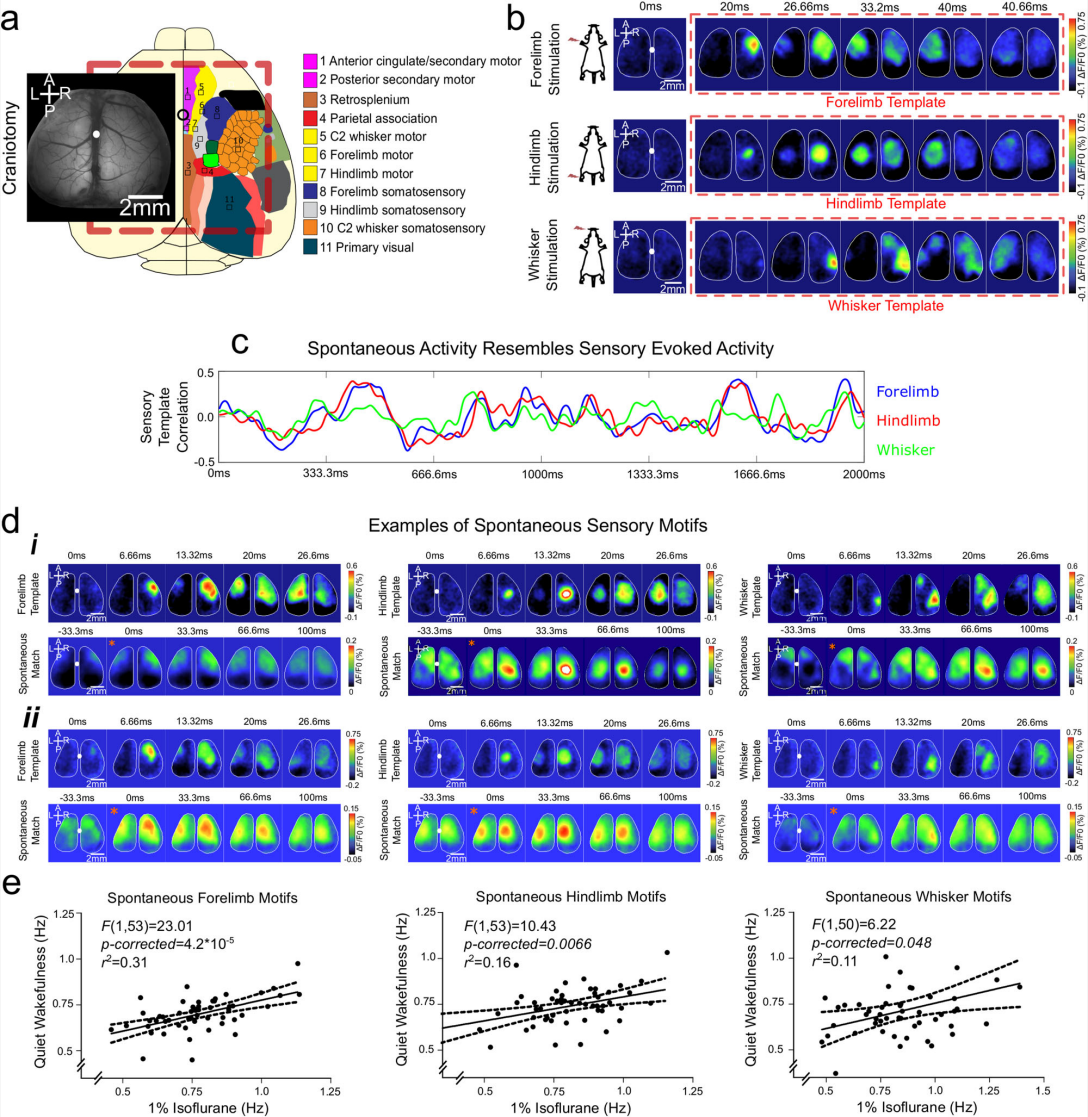
Sensory motifs in spontaneous cortical activity. **a** A representative craniotomy with VSD incubation and the mesoscale field of view alongside a schematic brain. **b** Standardized stimulation protocols yield sensory VSD responses with millisecond timescale resolution. **c** A montage of the correlation time course between spontaneous cortical activity and sensory derived templates. **d** (**i**–**ii**) Examples of sensory-evoked templates and spontaneous sensory motifs ‘matches’ from two animals. **e** The frequency of motifs in *n* = 53 animals recorded the anaesthetized sate is associated with the frequency of motifs during states of quiet wakefulness for forelimb (*F*(1,53) = 23.01, *p* = 1.4 × 10^−5^, *p*-corrected = 4.2 × 10^−5^), hindlimb (*F*(1,53) = 10.43, *p* = 0.0022, *p*-corrected = 0.0066), and whisker (*F*(1,50) = 6.22, *p* = 0.016, *p*-corrected = 0.048). Each point represents an individual animal and the frequency of spontaneous motifs identified over 50,005 frames of spontaneous activity in both conditions.

We performed in vivo voltage sensitive dye (VSD) imaging using an acute surgical preparation^18,22,23^. Mice underwent a craniotomy under isoflurane (1.0–1.5%) with buprenorphine (0.05 mg/kg intraperitoneally). Body temperature was maintained at 37 °C using a heating pad with a feedback thermistor. The skull was exposed and fastened to a steel plate. We performed a large 7 × 8 mm craniotomy (bregma 2.5 to −4.5 mm, lateral 0–4 mm) overlying both cortical hemispheres, and removed the underlying dura (Fig. 1a). We utilized RH-1692 dye (Optical Imaging, New York, NY) that was dissolved in HEPES-buffered saline solution (1 mg/mL). RH-1692 was incubated over the cortex for 60–90 min to allow the staining of all neocortical layers. Unbound RH-1692 was washed prior to covering the surface of the brain with 1.5% agarose made in HEPES-buffered saline and a glass coverslip.

### VSD image acquisition

Animals were imaged under 1.0% isofluorane with body temperature maintained at 37 °C. A subset of animals were imaged during quiet wakefulness. We captured 12-bit images with a charge-coupled device camera (1M60 Pantera, Dalsa, Waterloo, ON) and an EPIX E4DB frame grabber with XCAP 3.1 imaging software (EPIX, Inc., Buffalo Grove, IL). The focus was 800 μm below the surface to reduce signal distortion due to large cortical blood vessels. Images were taken through a macroscope composed of front-to-front video lenses (8.6 × 8.6 mm field of view, 67 μm per pixel).

We utilized a red LED (Luxeon, 627 nm) and 630 ± 15 nm filters. VSD fluorescence was filtered using a 673–703 nm bandpass optical filter (Semrock, New York, NY). To reduce regional bias in VSD signal caused by uneven dye loading or brain curvature, all VSD responses were expressed as a percentage change (Δ*F*/*F*_0_ × 100%) using Matlab (Mathworks, Natick, MA). VSD fluorescence was temporally filtered in Matlab using a zero-phase lag Chebyshev bandpass filter (zero-phase filter) at 0.5–6 Hz.

### Sensory-evoked responses

Sensory-evoked responses were quantified by calculating the average fluorescence within a 5 × 5 pixel region of interest centred on the maximally responsive pixel in MATLAB (MathWorks). For sensory-evoked variability, we utilized the peak response in the first ten frames following stimulation.

### Limb and whisker stimulation for individualized sensory templates

We presented ten sensory stimuli to forelimb, hindlimb, and whisker in order to obtain individualized templates of sensory experience. Limb stimulation involved subcutaneous acupuncture needles and 1 mA 1 ms electrical stimulation. For whisker stimulation, we utilized a piezoelectric bending actuator attached to the C2 whisker that achieved a 1-ms 0.6° displacement. In order to account for dye bleaching, responses to sensory stimuli were calculated as the normalized difference to the average of five stimulus-free trials (% Δ*F/F*_0_) in MATLAB (MathWorks).

### Spontaneous sensory motifs

As we have previously described^18^, we calculated the correlation between an animal’s sensory-evoked template and spontaneous cortical activity. The five-frame initial segment of sensory-evoked responses, limited to the hemisphere in which the primary response occurs (due to asymmetric evolution), was reshaped into a one-dimensional array and used as a template (Fig. 1b). We similarly reshaped spontaneous activity data, such that each index frame can be expressed as a correlation value to the sensory-evoked template.

To derive a correlation threshold for sensory motifs, we isolated template ‘matches’ at progressively higher thresholds to determine the quality of the ‘match’ (Fig. S1). We calculated the correlation coefficient between the template and each frame for the entire sample of spontaneous activity. This resulted in normal distribution of correlation values centred at zero-value correlation. We then isolated ‘matches’ in steps of 0.05 SD, and quantified the concordance between template and ‘match’ (Fig. S1). We decided to utilize the most distinctive frame of the template, the first frame, and the first frame of the ‘match’ (i.e. the first frame crossing the threshold) for concordance. We dichotomized the pixel values based on a threshold relative to the pixel with maximal Δ*F*/*F*_0_ in the frame. The threshold for dichotomization was qualitatively determined, and a threshold of 0.4 Δ*F*/*F*_0_ of the maximal pixel intensity within the frame isolated the spatial characteristics that were unique to sensory-evoked templates. Spontaneous ‘matches’ have lower peak Δ*F*/*F*_0_ values than sensory-evoked events, and therefore required a higher threshold to isolate spatial characteristics. These were 0.5 of maximal Δ*F*/*F*_0_ for whisker, 0.6 of Δ*F*/*F*_0_ for forelimb, and 0.7 of Δ*F*/*F*_0_ for hindlimb. These images allowed us to quantify the concordance of the template and match revealing a plateau in concordance at mean correlation + 1.5 SD.

For analyses quantifying spontaneous sensory motif frequency, a spontaneous sensory motif was deemed to have occurred when the template correlation exceeded this threshold for a minimum sequence of five frames. When examining the effect of spontaneous sensory motifs on sensory reliability, we also considered a continuous expression of spontaneous sensory motifs using template correlation values. We utilized both dichotomous and continuous approaches as differing motif thresholds principally differ in the magnitude of spontaneous activity within primary sensory regions (Fig. S1). Indeed ‘matches’ occurring at a lower threshold retain stereotyped features that could influence the representation of sensory events.

### Chronic social defeat

Four-month-old male CD1 mice were residents, into whose cage 8-week-old male C57BL/6J mice were introduced. There, they experienced physical defeat for 10 min, were separated by a perforated Plexiglas divider, and maintained in sensory contact overnight. For 10 days, mice experienced physical defeat by a new resident mouse^24^. Control mice were maintained separated by a divider and rotated daily without experiencing physical defeat.

### Maternal deprivation

C57BL/6J dams were removed from the home cage from P0–21 for 180 min per day^25^. Pups were placed within a temperature controlled (34 °C) humidified incubator. Control mice were facility-reared without separation.

### ChR2 circuitry manipulation

Using Thy1-ChR2 transgenic animals, we stimulated the anterior cingulate (0.7 mm anterior and 0.3 mm lateral to bregma) to manipulate circuitry involved in sensation and depressive-like behaviour as described by Barthas et al.^26^. We utilized a 1-ms 5-mW pulse generated by a 473-nm diode pumped solid-state laser (CNI, Optoelectronics) to stimulate ChR2 expressing neurons. As a control, we targeted primary somatosensory cortex. Each day over four consecutive days, 120 trains of 9600 pulses were delivered at 20 Hz with 2 s inter-train interval.

### Forced swim test

Mice were placed in a transparent glass beaker (25 cm height, 18 cm diameter), containing water at 24–5 °C. For 5 min, the mice remained in the water while an observer coded their active swimming and floating (including efforts to maintain position)^27^. The water was changed between animals. Only the last 4 min of the experiment are reported.

### Sucrose preference test

Mice were single-housed without prior food or water deprivation. They were habituated to the presence of two bottles for 24 h, which were replaced with fresh pseudoramdonly ordered bottles containing water or a 1% sucrose solution for 16 h. The sucrose preference was calculated as a ratio of the sucrose solution to water consumed.

### Drugs

Citalopram hydrobromide (Sigma-Aldrich) was stored in dimethyl sulfoxide (DMSO) stock solution and diluted in HEPES buffered saline (10 mg/kg, final DMSO concentration 0.05%). Vehicle injections were matched for DMSO concentration. To ensure that all behaviour and imaging findings were dissociated from the acute effects of the treatment, behaviour and imaging occurred 5 days following the completion of the stress protocol.

### Generation of null-hypothesis (‘shuffled’) templates and spontaneous activity

The two-dimensional fast Fourier transform (fft) of experimentally acquired sensory templates and spontaneous activity was multiplied by the fft of random grey values. Then, using the inverse two-dimensional fft, this was returned to the spatial domain resulting in simulated data for which the histogram of grey values, and the first- and second-order characteristics of the image are identical to the experimental data.

We generated 1000 null-hypothesis templates for *n* = 5 animals from forelimb, hindlimb, and whisker sensory responses, respectively. We correlated these templates with 50,005 frames of experimentally acquired spontaneous activity, generating 1001 (1 experimentally derived and 1000 simulated) correlation time courses for each modality. Experimental and null-hypothesis correlation distributions were compared using the Kolmogorov–Smirnov test.

For *n* = 11 epochs of spontaneous activity of 10,001 frames, we generated 10 null-hypothesis spontaneous activity datasets. We then calculated the correlation course with experimentally derived sensory templates. The experimental and null-hypothesis correlation distributions were compared using the Kolmogorov–Smirnov test.

### Viral injection

Viral delivery of the red shifted opsin, ChrimsonR^28^ (AAV9.Syn.ChrimsonR-tdTomato.WPRE.bGH, Penn Vector Core), involved syringe infusion pump injection (UMC4; World Precision Instruments). At 7 weeks of age, mice were injected with 1 µL to a depth of ~350 µm and speed of 1 nL/s. Under 1.5% isofluorane, injections were performed at three sites: (1) motor cortex (1 mm anterior and 2 mm lateral to bregma), (2) somatosensory cortex (1.5 mm posterior and 2.5 mm lateral to bregma), and (3) visual cortex (3.5 mm posterior and 2 mm lateral to bregma).

### Manipulating regional activity prior to sensory stimuli

We performed optical stimulation using the red-shifted opsin ChrimsonR^28^ in Ai85 animals, as we have previously described^15^. We utilized LED illumination (Luxeon, 470 nm) and a 467–499 nm excitation filter. iGluSnFR fluorescence emission was filtered using a 510–550 nm bandpass filter (Chroma). We limited temporal resolution to 50 Hz to mitigate 470 nm excitation and iGluSnFR-ChrimsonR crosstalk^15^.

We focused on barrel cortex and mapped both left and right C2 whisker responses (five piezo deflections, 100 Hz). Using these coordinates, we optically stimulated the right barrel cortex using a diode pumped solid-state laser delivering a 589-nm 200-µm diameter laser beam (CNI, Optoelectronics), and deflected the right C2 whisker at varying intervals after the laser pulse. We delivered 30 trials at laser-piezo intervals of 250 ms, 500 ms, 1000 ms, 1500 ms, 2000 ms, 5000 ms, and 7500 ms. Peak response amplitude was calculated using a 335 µm × 335 µm region of interest centred on the initial sensory response during mapping, and we subtracted the mean of the five frames preceding sensory stimulation.

### Statistics

Samples sizes for group and sex were determined as 6–8 based on our previous experience with these techniques. Sex-based analyses were not pre-planned. For combined optical stimulation and imaging experiments, we conservatively estimated a moderate effect size of 0.5, and for seven within subject samples we determined that a sample of four animals would have 0.95 power with *α* ≤ 0.05. We utilized the Student’s *t*-test or ANOVA (with Dunnet post-hoc comparison) for normally distributed data. We utilized the Mann–Whitney test for non-normally distributed data. Linear regression was utilized to test the relationships between depressive-like behaviour and summary statistics from imaging. After testing for normality with the Shapiro Wilks test, we utilized generalized linear mixed effect models (GLMEM; normal distribution and identity link function) to examine the effect of spontaneous sensory motifs and the fidelity of sensory responses to allow a subject random effect. We utilized the Kolmogorov–Smirnov test to compare the population correlation distribution differences. Significance was set at *α* ≤ 0.05, and we applied Bonferroni correction for non-independent analyses. All analyses were performed using MATLAB (MathWorks) and plots generated with MATLAB or PRISM 5 (GraphPad).

## Results

Utilizing an acute surgical mesoscale preparation together with VSD (Rh1692, Optical Imaging) and millisecond timescale imaging (Movie S1; Fig. 1a), we characterize spontaneous cortical motifs in murine models of stress. Sensory responses and spontaneous epochs totalling 50,005 frames (150 Hz) were recorded under light isoflurane anaesthesia (1%) to ensure a state devoid of potential motor planning, sensory contaminants, or fear behaviours. Spontaneous recordings were also conducted in a state of quiet wakefulness for a subset of animals.

As spontaneous activity dynamics can change transiently after sensory stimulation to overrepresent recent sensory activity^19^, only after acquiring spontaneous activity did we utilize standard peripheral stimulation protocols for subcutaneous electrical stimulation to forelimb and hindlimb as well as mechanical deflection of the C2 whisker (Fig. 1b). These, in turn, were utilized as templates to describe activity dynamics in the preceding spontaneous activity acquisition.

The time course of these template-correlation values within spontaneous activity revealed a distribution of positive and negative correlation values, with distinctive positive inflections and peaks (Fig. 1c). Examples of spontaneous template matches from two animals are presented in Fig. 1d, illustrating the importance of an individualized template despite the identification of broadly consistent sensory motifs. Though our correlation analyses are limited to a single hemisphere, the spontaneous motifs identified are bilateral.

We sought to ensure that the frequency of motifs occurring in the anaesthetized state, chosen to reduce the effects of sensorimotor planning or sensory contamination, could inform cortical dynamics in wakefulness. We therefore compared the frequency of motif occurrence in spontaneous activity epochs recorded under 1% isoflurane and under quiet wakefulness from *n* = 53 animals (Fig. 1e). Linear regressions revealed statistically significant relationships between the frequency of motifs occurring under anaesthesia and in a state of quiet wakefulness, though the absolute frequency was reduced during quiet wakefulness for all three modalities.

As it is possible that limb and whisker motifs are simply exemplars among a diverse palette of activity patterns in dorsal neocortex, or alternatively a global increase in power in the frequency domain we characterize, we sought to generate alternative templates to explore the relative importance of other patterns excluded by our strategy. Null-hypothesis, or ‘shuffled’, templates and spontaneous activity were generated by altering the spatial domain of experimentally acquired data, while preserving the grey-value histograms, as well as first- and second-order moments (Fig. S2a–d). We performed 1000 template shuffles for each modality, and found that the frequency of motif matches using sensory-evoked templates lay outside the 99% confidence interval of the null-hypothesis data (see Fig. S2B for single animal examplar), and that the correlation values between ‘shuffled’ templates and experimental (‘real’) spontaneous data revealed an overrepresentation of zero or near zero correlations (Fig. S2C; *n* = 5 animals). We repeated this process shuffling 10,000 frame epochs of spontaneous data (Fig. S2E, ten shuffles per animal), and found that motif matches in experimental data exceeded those in shuffled data (see Fig. S2F for single animal exemplar), with an overrepresentation of zero or near-zero correlations in the ‘shuffled’ spontaneous data (Fig. S2G; *n* = 10 animals). Thus, the spatiotemporal organization captured by sensory templates are well-represented spontaneous fluctuations within the field of view.

### Sensory motifs are stress-susceptible

**Fig. 2:**
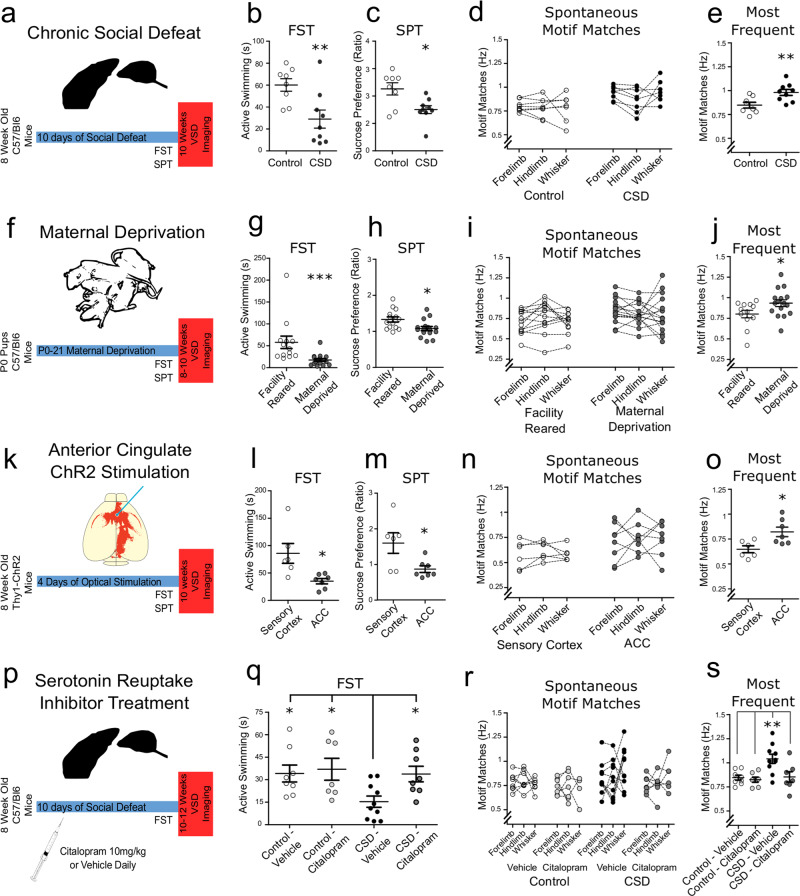
Stressed animals have an increased frequency of spontaneous sensory motifs. **a** A schematic representation of CSD model of stress in male animals. Defeated animals exhibited **b** decreased active coping on the FST (*t*(15) = 3.02, *p* = 0.0085, *p*-corrected = 0.017) and **c** decreased hedonic response according to the SPT (*t*(15) = 2.39, *p* = 0.030, *p*-corrected = ns). **d** Spontaneous cortical motif matches were increased for forelimb (*t*(12) = 2.47, *p* = 0.029, *p*-corrected = ns) and whisker templates (*t*(14) = 4.19, *p* = 0.0009, *p*-corrected = 0.0036), but not hindlimb templates (*t*(13) = 1.50, *p* = 0.15, *p*-corrected = ns). **e** Isolating the most represented motif identified upregulation in defeated animals (*t*(15) = 3.03, *p* = 0.0084, *p*-corrected = 0.033). **f** A schematic representation of the maternal deprivation early life model of stress (7F/10M deprived, 7F/6M facility reared). Deprived animals exhibited behavioural sequelae according to the FST (**g**) (sum of ranks 308 vs 157, *p* = 0.0002, *p*-corrected = 0.0004) and the SPT (**h**) (*t*(28) = 2.74, *p* = 0.010, *p*-corrected = 0.02) relative to facility-reared animals. **i** The frequency of spontaneous motifs were increased for whisker (*t*(26) = 2.79, *p* = 0.0096, *p*-corrected = 0.038), but not forelimb (*t*(26) = 1.57, *p* = 0.12, *p*-corrected = ns) or hindlimb (*t*(26) = 0.70, *p* = 0.48, *p*-corrected = ns). **j** Isolating the most represented motif identified upregulation in deprived animals (*t*(15) = 3.03, *p* = 0.0084, *p*-corrected = 0.033). **k** A representation of optogenetic stimulation of the anterior cingulate cortex (2F/5M ACC stimulated; 1F/5M sensory cortex stimulated). ACC stimulated animals exhibited behavioural sequelae according to the FST (**l**) (sum of ranks 62 vs 29, *p* = 0.0023, *p*-corrected = 0.0046) and the SPT (**m**) (sum of ranks 57 vs 34, *p* = 0.0381, *p*-corrected = ns) relative to sensory cortex stimulated animals. **n** Spontaneous sensory motif matches were not increased for forelimb (*t*(11) = 0.81, *p* = 0.43, *p*-corrected = ns), hindlimb (*t*(11) = 1.71, *p* = 0.11, *p*-corrected = ns), and whisker (*t*(11) = 1.03, *p* = 0.32, *p*-corrected = ns) templates. **o** Isolating the most represented motif identified upregulation in defeated animals (*t*(11) = 2.85, *p* = 0.015, *p*-corrected = 0.06). **p** A schematic representation of chronic social defeat and selective serotonin reuptake inhibitor treatment. **q** Citalopram treatment normalizes FST behaviour in defeated animals (*F*(3,32) = 3.84, *p* = 0.0196; Dunnett’s multiple comparison illustrated). **r** Spontaneous sensory motifs matches were not different between groups for forelimb (*F*(3,23) = 2.64, *p* = 0.073, *p*-corrected = ns), hindlimb (*F*(3,28) = 0.47, *p* = 0.70, *p*-corrected = ns), or whisker (*F*(3,28) = 0.77, *p* = 0.51, *p*-corrected = ns); however **s** isolating the most represented motif revealed an increase in motifs in vehicle-treated animals relative to all other groups (*F*(3,28) = 6.63, *p* = 0.0016, *p*-corrected = 0.0064; Dunnet’s multiple comparison illustrated). Error bars in graphs represent mean ± standard error. *p-corrected < 0.05, **p-corrected < 0.01. ***p-corrected < 0.001, ^#^*p* = 0.06.

To determine whether spontaneous sensory motifs are susceptible to stress, we first examined the CSD stress model (Fig. 2a). Given our simultaneous interest in sensation, we had a very low threshold for experimenter intervention during defeats to prevent injuries and their related confounds. Daily sessions were not shortened to achieve this, instead the interaction was interrupted before allowing it to resume if injury appeared imminent. Animals sustaining visible wounds of any size were excluded and not imaged. We observed behavioural sequelae in stressed animals according to the forced swim test (FST) and sucrose preference test (SPT) (Fig. 2b, c). When we examined correlations between sensory templates and spontaneous activity, we observed broadened correlation frequency distributions and thus a higher prevalence of spontaneous activity highly correlated with sensory templates in stressed animals (Fig. S3Ai–vi). When we examined motif matches, forelimb and whisker motifs occurred at a higher frequency in stressed animals (Fig. 2d). We noted that motifs were not uniformly upregulated in stressed animals, as supported by a lower intraclass correlation relative to control animals (ICC = 0.61 control animals, *p* = 0.06; ICC = 0.51 defeated animals, *p* = 0.12). Yet, in all cases, at least one motif was upregulated (Fig. 2e).

We sought to confirm our finding in a model without physical subjugation. We therefore turned to the MD early life stress model (Fig. 2f). When these animals were tested in adulthood, behavioural differences were observed on the FST and the SPT (Fig. 2g, h). A broader population distribution of correlation values in the maternally deprived group was observed for all modalities (Fig S3Bi–vi), consistent with persistent alterations to cortical dynamics and behaviour after early life stress. In this model, only forelimb matches were increased (Fig. 2i). Once again there appeared to be an uneven upregulation of motifs in stressed animals (intra-class correlation (ICC) = 0.77 facility reared, *p* < 0.001; ICC = 0.66 MD, *p* = 0.005), and by isolating the most frequently occurring motif, maternally deprived animals had an increased motif frequency relative to facility-reared animals (Fig. 2j).

These models, however, rely on sensory inputs and impact numerous organ systems^29^. Therefore, we sought to directly manipulate brain networks implicated in sensory integration and emotional behaviour, notably the anterior cingulate cortex (ACC)^26,30^. With the Thy1-ChR2 transgenic mouse, we delivered trains of optical stimulation to the ACC^26^, or primary sensory cortex as a control intervention (Fig. 2k). When tested 10 days later, ACC-stimulated animals exhibited alterations on the FST and SPT (Fig. 2l, m). ACC-stimulated animals exhibited broadened population correlation distributions relative to sensory-cortex-stimulated animals (Fig. S3Ci–vi), mimicking the circuit level phenomenon observed after stress. No individual motif was upregulated in this group (Fig. 2n); however, once again we observed lower inter-motif frequency consistency (ICC = 0.84, *p* = 0.01, sensory stimulated; ICC = 0.67, *p* = 0.04, ACC stimulated) in the ACC-stimulated group, and isolating the maximally expressed motif revealed a higher frequency in ACC relative to control animals (Fig. 2o). These results are consistent with the observed circuit level changes being sufficient to re-capitulate a stress phenotype.

We next tested whether selective serotonin reuptake inhibitor treatment would prevent altered spontaneous sensory dynamics resulting from chronic stress. We repeated the CSD protocol in conjunction with daily administration of a commonly utilized selective serotonin reuptake inhibitor, citalopram (10 mg/kg, intraperitoneal), or vehicle beginning on the first day of the CSD procedure (Fig. 2p). After a 5-day washout period, citalopram-treated defeated animals exhibited normalized FST behaviour (Fig. 2q). Sensory motifs were upregulated in vehicle-treated defeated animals, and we observed a low intra-class correlation relative to the other groups (Fig. 2r; ICC = −0.48, *p* = 0.72 CSD vehicle animals; ICC = 0.58, *p* = 0.007 other groups). Isolating the most represented spontaneous motif revealed increased an increased frequency in vehicle-treated defeated animals, whereas citalopram-treated CSD animals did not differ from control groups (Fig. 2s).

### Sensory motifs and active coping behaviour

**Fig. 3:**
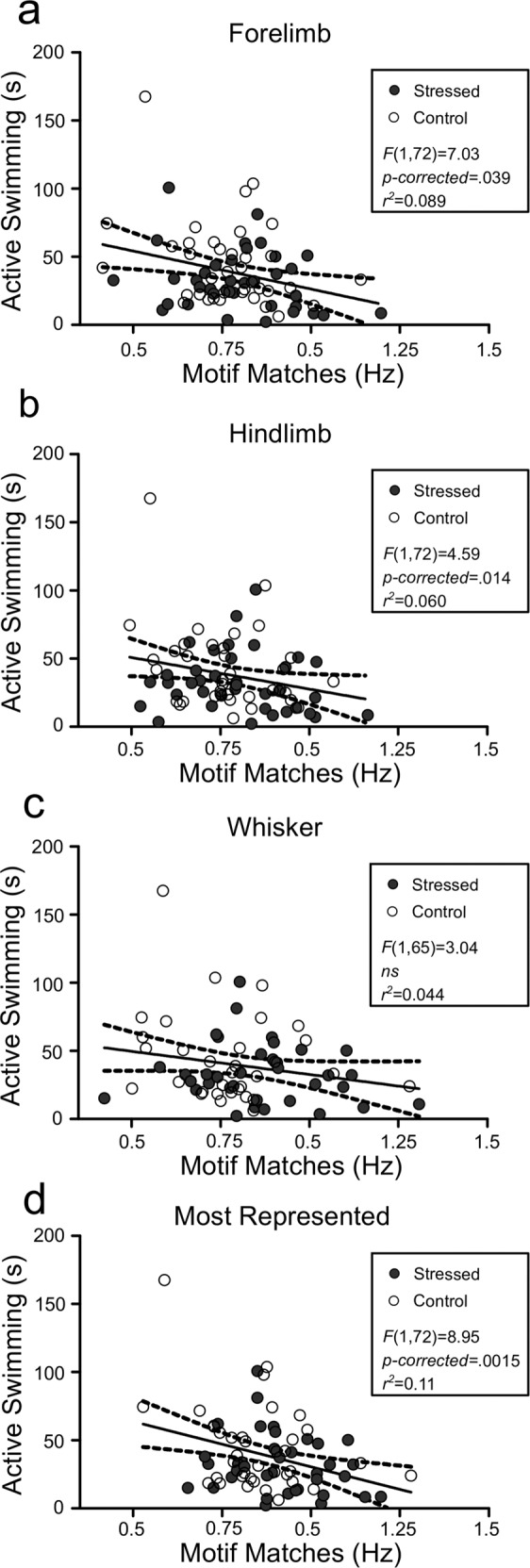
Spontaneous sensory motif frequency is related to active coping behaviour. **a** Forelimb spontaneous motif frequency was negatively related to active coping on the FST (*F*(1,72) = 7.03, *p* = 0.0098, *p*-corrected = 0.039). **b** Hindlimb spontaneous motif frequency was also negatively related to active coping on the FST (*F*(1,72) = 4.59, *p* = 0.0035, *p*-corrected = 0.014), while **c** whisker spontaneous motif frequency not significantly related to active coping on the FST (*F*(1,65) = 3.04, *p* = 0.085, *p*-corrected = ns). Isolating the motif with maximal frequency revealed a strong negative anticorrelation with active coping on the FST (*F*(1,72) = 8.95, *p* = 0.00038, *p*-corrected = 0.0015).

Although there were cohort effects evident in active coping behaviour, we sought to test whether motif frequency was related to the behavioural sequelae of our manipulations. In males, we observed modest but statistically robust relationships between the frequency of limb and whisker motifs (Fig. 3a–c), as well as the individual animal’s most frequent motif (Fig. 3d), and active coping on the FST. We did not observe this in females where there was substantial variability (Fig. S4), possibly due to the oestrous cycle and delays between behaviour and imaging.

### Stress increases the variability in sensory responses in proportion to motifs

**Fig. 4:**
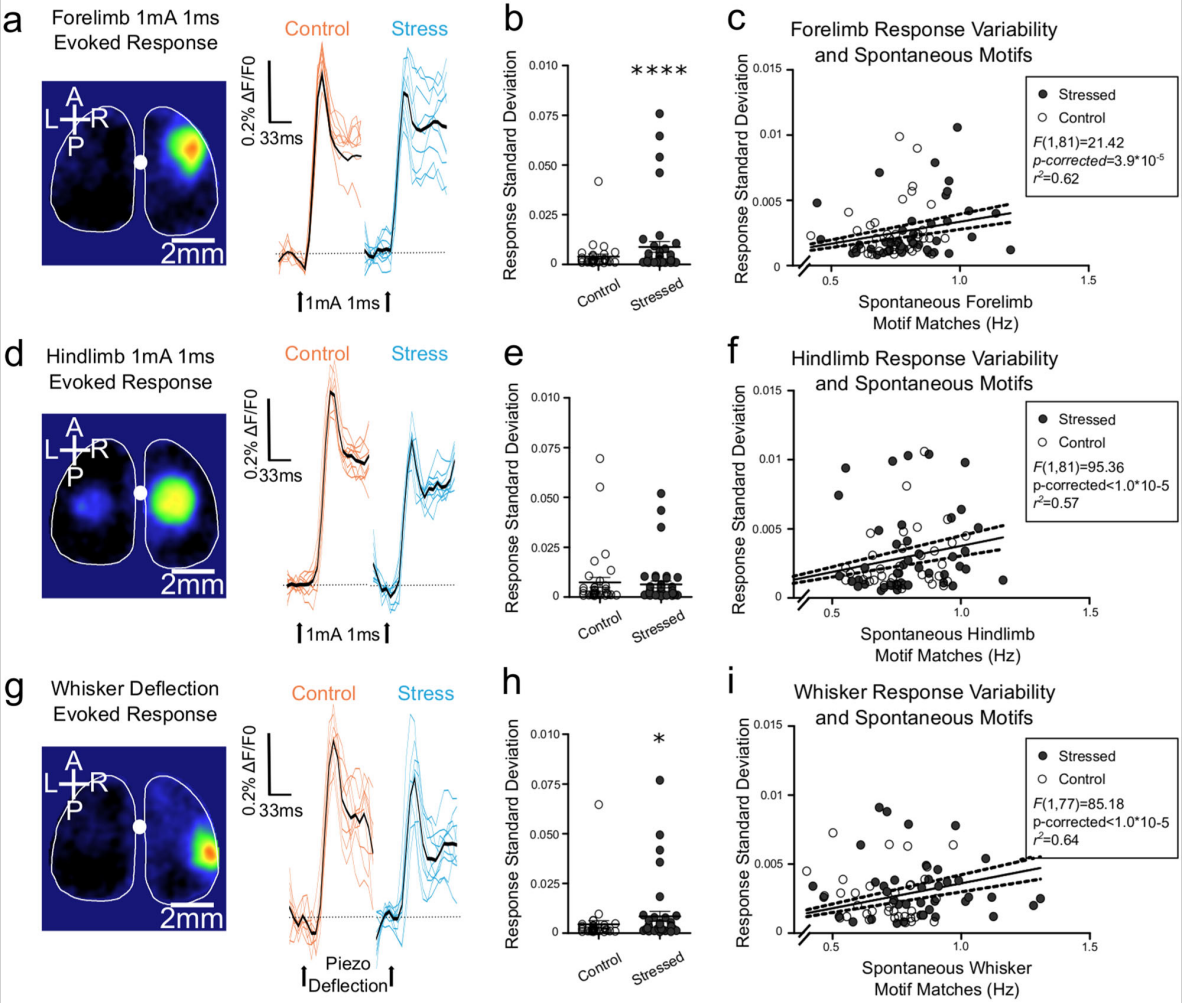
Variability in sensory-evoked responses is impacted by stress and is related to the frequency of spontaneous sensory motifs. **a** A representative VSD image of forelimb response to electrical stimulation and traces of evoked responses for control (orange) and stressed (blue) animals. **b** Stressed animals had increased variability in their evoked response standard deviations (*F* = 6.05, *p* < 0.0001). **c** Variability in forelimb responses to electrical stimulation was linearly related to the frequency of forelimb motifs captured during spontaneous activity sampling prior to stimulation trials (*F*(1,88) = 21.42, *p* = 1.3 × 10^−5^, *p*-corrected = 3.9 × 10^−5^). **d** A representative VSD image of hindlimb response to electrical stimulation and traces of evoked responses. **e** Stressed and control animals had comparable variability in sensory-evoked responses (*F* = 0.08, ns); however, **f** variability in responses was linearly related to the frequency of spontaneous hindlimb motifs during spontaneous activity (*F*(1,81) = 95.36, *p* < 0.00001, *p*-corrected < 0.00001). **g** A representative VSD image of whisker piezo deflection and traces of evoked responses. **h** Variability in whisker responses was increased in stressed animals (*F* = 2.05, *p* = 0.037), and **i** variability was linearly related to the frequency of spontaneously occurring whisker motifs (*F*(1,77) = 85.18, *p* < 0.00001, *p*-corrected < 0.00001). For linear regressions, outliers were identified using Tukey’s Fences and excluded. Error bars in graphs represent mean ± standard error.

Qualitatively, stressed animals had greater variability of the initial sensory responses than control animals (Fig. 4a, d, g). We quantified this using standard deviation of sensory-evoked responses. Forelimb responses and whisker responses were significantly more variable in stressed animals (Fig. 4b–h), but not hindlimb responses (Fig. 4e). These distributions, however, had notable outliers in both stressed and control animals. As our hypothesis is that sensory motifs preceding sensory events impacts sensory variability, we performed linear regressions to test the spontaneous frequency of sensory motifs and sensory variability. As there were more extreme outliers in the stressed group, we wished to ensure that these were not driving effects, and therefore they were excluded using Tukey’s Fences (>0.0125; forelimb *n* = 7 excluded, hindlimb *n* = 9 excluded, whisker *n* = 7 excluded). These regressions revealed that the frequency of motif occurrence in the period of spontaneous activity recorded prior to sensory stimulation was associated with variability across all three modalities (Fig. 4c, f, i).

### Spontaneous cortical motifs impact sensory responses

**Fig. 5:**
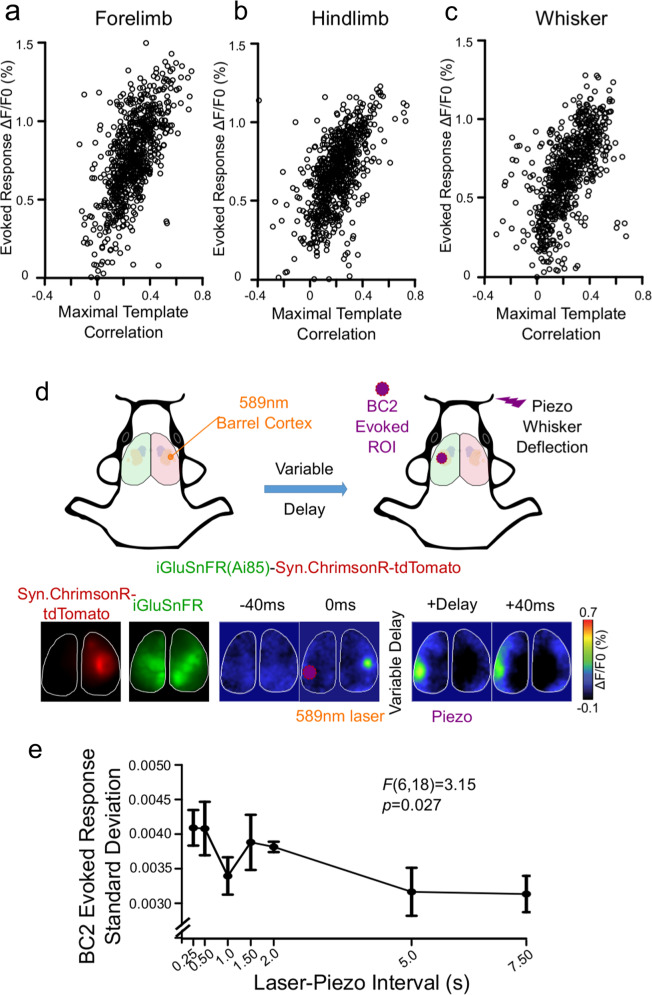
Motifs and regional activity preceding sensory-evoked responses influence response variability. **a**–**c** Scatterplots illustrating the relationship between sensory patterned activity preceding stimulation and the corrected amplitude of sensory responses to electrical subcutaneous limb stimulation or piezoelectric whisker deflection (ten trials per modality per animal, *n* = 77 animals). The 200 ms baseline for each trial was correlated with the sensory template, and the maximal correlation isolated. Generalized linear mixed effect models with random effects for individual mice revealed significant relationships between maximal correlation and response amplitude for **a** forelimb responses (estimate = 0.52, 95%CI: 0.40–0.63, *t*(768) = 8.81, *p* = 8.24 × 10^−18^, *p*-corrected = 2.47 × 10^−17^), **b** hindlimb responses (estimate = 0.41, 95%CI: 0.31–0.51, *t*(768) = 8.08, *p* = 2.49 × 10^−15^, *p*-corrected = 7.47 × 10^−15^), and **c** whisker responses (estimate = 0.50, 95%CI: 0.41–0.59, *t*(768) = 11.22, *p* = 3.41 × 10^−27^, *p*-corrected = 1.02 × 10^−26^). **d** A schematic representation of the experimental strategy where activity in barrel sensory cortex was optically induced with ChrimsonR and a 589-nm laser pulse, and after a variable interval (250 ms–7.5 s) the C2 whisker was piezo deflected. Below the schematic are representative images taken in vivo from the experiment visualizing Syn.ChrimsonR-tdTomato with 525 nm excitation and 645 nm emission filters, iGluSnFR with 470 nm excitation and 535 nm emission filters, alongside a montage illustrating the response ROI, the 589 nm laser response, and the sensory-evoked response after a variable delay. **e** The standard deviation of 30 trials delivered at each interval revealed increased variability as the laser pulse-piezo interval narrowed (*n* = 4 animals, *F*(6,18) = 3.15, *p* = 0.027). Error bars in graph represent mean ± standard error.

We next characterized how spontaneous sensory motifs impact sensory-evoked responses on a trial to trial basis. As differing thresholds for the isolation of motifs principally differ in the magnitude of spontaneous activity within primary sensory regions (Fig. S1), a dichotomous characterization of motifs is insensitive to the effects of spontaneous cortical dynamics on cortical representations of sensory events. We therefore focused on the template correlation values to characterize the spontaneous activity preceding each trial. To prevent additive effects of spontaneous fluctuations and responses, response amplitude was corrected for the mean Δ*F*/*F*_0_ signal in the 20 ms (three frames) preceding the initial sensory response^6,7^. As our hypothesis is informed by the established relationship between cortical state and sensory responses^5,7^, we first isolated the sensory template correlation at the moment of the sensory stimulus and tested its relationship to sensory-evoked response magnitude using General Linear Mixed Effects Modelling (GLMEM). Contrary to our hypotheses, we found no relationship between sensory template correlation at stimulus delivery and response amplitude. When we isolated the maximal sensory template correlation within the 200 ms baseline preceding each stimulation trial, GLMEM (Wilkinson notation: Sensory_response ~ Maximal_template_correlation + (1|Mouse)) identified a positive association with the magnitude of sensory responses (Fig. 5a–c).

We next sought to experimentally manipulate the time interval between cortical sensory motifs and sensory stimuli to characterize the temporal effects of spontaneous sensory motifs on cortical sensory representaitons. For these experiments, we utilized the Ai85 mouse^20^ expressing the extracellular glutamate sensor iGluSnFR^31^, with similar temporal kinetics to VSD^21^, in conjunction with Syn-ChrimsonR^28^. This approach was chosen over optical stimulation with VSD to avoid phototoxicity. Due to weak cross-talk between iGluSnFR and ChrimsonR (Fig. S5), we elicited and quantified sensory responses in the hemisphere contralateral to the ChrimsonR driven activity (Fig. 5d) as we have previously shown that unilateral optical stimulation evokes bilateral VSD motifs^32^. We acquired 30 sensory responses at multiple time intervals between laser stimulation of barrel cortex and whisker piezo deflection. Sensory response magnitude in the 5 × 5 pixel ROI of the primary sensory response was corrected to spontaneous Δ*F*/*F*_0_ in the five frames preceding stimulation. As the interval between ChrimsonR depolarization in barrel cortex and whisker deflection narrowed, sensory-evoked responses became more variable (Fig. 5e), indicating that regional depolarization motifs can impact sensory reliability for seconds.

## Discussion

Neural circuitry does not reproducibly respond in the same way to the same stimulus, and our data support the hypothesis that stress reduces the reliability of sensory-evoked responses through altered cortical activity dynamics. Stress does not uniformly increase the frequency of spontaneous activity motifs in somatosensory cortex. The affected motif is idiosyncratic, or unique to the animal, rather a global increase in activity. In line with past findings examining localized spontaneous depolarizations in somatosensory cortex^5,7^, we show that spontaneous sensory motifs are associated with variability in sensory-evoked responses. Moreover, by optically manipulating cortical activity, we show that regional depolarization has a persistent effect on sensory reliability. Our data suggest that spontaneous sensory motifs may provide an assay to understand sensory phenotypes associated with stress and emotional states.

Cortical waves of activity resembling the spontaneous motifs we characterize are described in the context of sensory-evoked events, where thalamocortical inputs drive an initial response that then propagates via horizontal connections^33,34^. These long-range intracortical connections indicate recurrent interactions in cortex^35^. Cortical waves are predominantly composed of subthreshold activity, to which VSD is well suited; however, delayed spiking has also been observed distal to the immediate thalamic projections^36^. Similar to spontaneous motifs^18^, stimulus-evoked cortical activity waves propagate across functional boundaries^33^. The function of sensory-evoked cortical waves remains unclear; however, they have been proposed to contribute to sensory processing by retaining temporal information relating to stimulus onset^34^. They have furthermore been proposed to improve sensory contrast through phasic facilitation and suppression of sensory information^33^.

While spontaneous motifs may serve these same functions, they do not depend on sensory input and continue to occur in somatosensory cortex that has been peripherally deafferented^8^. Similarly, our experimental strategy employed multiple models that relied progressively less on sensory inputs (social defeat, maternal isolation, and ACC optical stimulation), but nevertheless demonstrated convergent validity of an emotional phenotype, spontaneous motifs, and sensory-evoked variability. While a purely corticocortical phenomenon is possible^37^, thalamocortical and other subcortical-cortical circuits are likely^38,39^. An additional possibility is that spontaneous cortical sensory motifs represent a phenomenon of sensory consolidation or replay of experience, as in hippocampal consolidation^40,41^.

Seminal studies on spontaneous activity and sensory variability using VSDs referred to network states^6^ and internal sensory representations^10^ upon which sensory processing is superimposed. Indeed, spontaneous activity may serve as a means of isolating salience through response facilitation or inhibition^33^ or enhancing responses to weak stimuli^42^. Interoceptive and self-monitoring functions of spontaneous activity^43^ play an important role in contextualizing sensory-evoked events^44,45^, and the relationship between emotionality and interoceptive inference has been previously postulated^12,45,46^.

The structure and changes in spontaneous cortical dynamics in model species and in human psychopathology will provide an avenue for further study and, potentially, therapeutics. Replicating this phenomenon in humans may be achieved with non-invasive electrophysiology, such as electroencephalography or magnetoencephalography. An analogous phenotype has been demonstrated using magnetoencephalography in patients with Complex Regional Pain Syndrome Type 1, highly relevant to our findings as this subtype occurs without nerve injury to account for changes. Compared to healthy controls, patients had increased resting state spectral power in delta and theta frequencies, and utilizing independent component analysis this was isolated to affective and somatosensory regions corresponding to the individual-specific somatic complaint^47^. We speculate that this approach integrates the subjective experience of the individual and functional neural substrates, with applicability to other conditions characterized by unexplained somatic symptoms.

Yet, our findings appeared specific to male animals and we did not observe the same strength of association between stress and spontaneous sensory motifs in female animals. This is an important limitation of our findings and merits highlighting as somatic symptom disorders are equally prevalent in males and females with conditions such as depression^48^, and given a higher prevalence of such conditions among females, the population prevalence of somatization is higher in females. Additional approaches, such as gonadectomized animals, may be required to understand the high degree of variance in motif frequency we observed in females.

Succinctly, patients with depression commonly seek medical attention for physical complaints with no identifiable causes^1,49^, and there is sufficient evidence that this is not simply an ‘idiom of distress’^4,50^. Using convergent lines of evidence in mouse, we implicate spontaneous sensory motifs as a stress-sensitive determinant of sensory reliability, with potential implications for psychiatric conditions and functional neurological syndromes.

## Supplementary information

Video 1.

Supplemental Figure legends

SFig 1.

SFig 2.

SFig 3.

SFig 4.

SFig 5.

## Data Availability

All data associated with the study are available in the main text or the supplementary materials. Computer code is available upon reasonable request.

## References

[1] Simon GE, VonKorff M, Piccinelli M, Fullerton C, Ormel J (1999). An international study of the relation between somatic symptoms and depression. N. Engl. J. Med..

[2] Moldofsky H, Scarisbrick P (1976). Induction of neurasthenic musculoskeletal pain syndrome by selective sleep stage deprivation. Psychosom. Med..

[3] Simon GE, Gureje O (1999). Stability of somatization disorder and somatization symptoms among primary care patients. Arch. Gen. Psychiatry.

[4] Klauenberg S (2008). Depression and changed pain perception: hints for a central disinhibition mechanism. Pain.

[5] Deneux T, Grinvald A (2016). Milliseconds of sensory input abruptly modulate the dynamics of cortical states for seconds. Cereb. Cortex.

[6] Arieli A, Sterkin A, Grinvald A, Aertsen A (1996). Dynamics of ongoing activity: explanation of the large variability in evoked cortical responses. Science.

[7] Petersen CC, Hahn TT, Mehta M, Grinvald A, Sakmann B (2003). Interaction of sensory responses with spontaneous depolarization in layer 2/3 barrel cortex. Proc. Natl Acad. Sci. USA.

[8] Poulet JF, Petersen CC (2008). Internal brain state regulates membrane potential synchrony in barrel cortex of behaving mice. Nature.

[9] Curto C, Sakata S, Marguet S, Itskov V, Harris KD (2009). A simple model of cortical dynamics explains variability and state dependence of sensory responses in urethane-anesthetized auditory cortex. J. Neurosci..

[10] Kenet T, Bibitchkov D, Tsodyks M, Grinvald A, Arieli A (2003). Spontaneously emerging cortical representations of visual attributes. Nature.

[11] Luczak A, Bartho P, Harris KD (2009). Spontaneous events outline the realm of possible sensory responses in neocortical populations. Neuron.

[12] Seth, A. K. & Friston, K. J. Active interoceptive inference and the emotional brain. *Philos. Trans. R Soc. Lond. B Biol. Sci.* **371**, 20160007 (2016). 10.1098/rstb.2016.0007 PMC5062097 28080966

[13] Gusnard DA, Raichle ME (2001). Searching for a baseline: functional imaging and the resting human brain. Nat. Rev. Neurosci..

[14] Sheline YI (2009). The default mode network and self-referential processes in depression. Proc. Natl Acad. Sci. USA.

[15] McGirr A, LeDue J, Chan AW, Xie Y, Murphy TH (2017). Cortical functional hyperconnectivity in a mouse model of depression and selective network effects of ketamine. Brain.

[16] Luczak A, Bartho P, Harris KD (2013). Gating of sensory input by spontaneous cortical activity. J. Neurosci..

[17] Luczak A, Bartho P, Marguet SL, Buzsaki G, Harris KD (2007). Sequential structure of neocortical spontaneous activity in vivo. Proc. Natl Acad. Sci. USA.

[18] Mohajerani MH (2013). Spontaneous cortical activity alternates between motifs defined by regional axonal projections. Nat. Neurosci..

[19] Han F, Caporale N, Dan Y (2008). Reverberation of recent visual experience in spontaneous cortical waves. Neuron.

[20] Madisen L (2015). Transgenic mice for intersectional targeting of neural sensors and effectors with high specificity and performance. Neuron.

[21] Xie Y (2016). Resolution of high-frequency mesoscale intracortical maps using the genetically encoded glutamate sensor iGluSnFR. J. Neurosci..

[22] Chan AW, Mohajerani MH, LeDue JM, Wang YT, Murphy TH (2015). Mesoscale infraslow spontaneous membrane potential fluctuations recapitulate high-frequency activity cortical motifs. Nat. Commun..

[23] Lim DH, LeDue JM, Mohajerani MH, Murphy TH (2014). Optogenetic mapping after stroke reveals network-wide scaling of functional connections and heterogeneous recovery of the peri-infarct. J. Neurosci..

[24] Golden SA, Covington HE, Berton O, Russo SJ (2011). A standardized protocol for repeated social defeat stress in mice. Nat. Protoc..

[25] MacQueen GM, Ramakrishnan K, Ratnasingan R, Chen B, Young LT (2003). Desipramine treatment reduces the long-term behavioural and neurochemical sequelae of early-life maternal separation. Int. J. Neuropsychopharmacol..

[26] Barthas F (2015). The anterior cingulate cortex is a critical hub for pain-induced depression. Biol. Psychiatry.

[27] Porsolt RD, Bertin A, Jalfre M (1977). Behavioral despair in mice: a primary screening test for antidepressants. Arch. Int. Pharmacodyn. Ther..

[28] Klapoetke NC (2014). Independent optical excitation of distinct neural populations. Nat. Methods.

[29] Soderholm JD (2002). Neonatal maternal separation predisposes adult rats to colonic barrier dysfunction in response to mild stress. Am. J. Physiol. Gastrointest. Liver Physiol..

[30] Vogt BA, Paxinos G (2014). Cytoarchitecture of mouse and rat cingulate cortex with human homologies. Brain Struct. Funct..

[31] Marvin JS (2013). An optimized fluorescent probe for visualizing glutamate neurotransmission. Nat. Methods.

[32] Lim DH (2012). In vivo large-scale cortical mapping using channelrhodopsin-2 stimulation in transgenic mice reveals asymmetric and reciprocal relationships between cortical areas. Front. Neural Circuits.

[33] Sato TK, Nauhaus I, Carandini M (2012). Traveling waves in visual cortex. Neuron.

[34] Muller L, Chavane F, Reynolds J, Sejnowski TJ (2018). Cortical travelling waves: mechanisms and computational principles. Nat. Rev. Neurosci..

[35] Wu JY, Xiaoying H, Chuan Z (2008). Propagating waves of activity in the neocortex: what they are, what they do. Neuroscientist.

[36] Bringuier V, Chavane F, Glaeser L, Fregnac Y (1999). Horizontal propagation of visual activity in the synaptic integration field of area 17 neurons. Science.

[37] Tsodyks M, Kenet T, Grinvald A, Arieli A (1999). Linking spontaneous activity of single cortical neurons and the underlying functional architecture. Science.

[38] Xiao, D. et al. Mapping cortical mesoscopic networks of single spiking cortical or sub-cortical neurons. *Elife* 6, e19976 (2017). 10.7554/eLife.19976 PMC5328594 28160463

[39] Hultman R (2018). Brain-wide electrical spatiotemporal dynamics encode depression vulnerability. Cell.

[40] Carr MF, Jadhav SP, Frank LM (2011). Hippocampal replay in the awake state: a potential substrate for memory consolidation and retrieval. Nat. Neurosci..

[41] O’Neill J, Pleydell-Bouverie B, Dupret D, Csicsvari J (2010). Play it again: reactivation of waking experience and memory. Trends Neurosci..

[42] Wiesenfeld K, Moss F (1995). Stochastic resonance and the benefits of noise: from ice ages to crayfish and SQUIDs. Nature.

[43] Fox MD, Snyder AZ, Zacks JM, Raichle ME (2006). Coherent spontaneous activity accounts for trial-to-trial variability in human evoked brain responses. Nat. Neurosci..

[44] Crapse TB, Sommer MA (2008). Corollary discharge across the animal kingdom. Nat. Rev. Neurosci..

[45] Barrett LF, Simmons WK (2015). Interoceptive predictions in the brain. Nat. Rev. Neurosci..

[46] Edwards MJ, Adams RA, Brown H, Parees I, Friston KJ (2012). A Bayesian account of ‘hysteria’. Brain.

[47] Walton KD, Dubois M, Llinas RR (2010). Abnormal thalamocortical activity in patients with Complex Regional Pain Syndrome (CRPS) type I. Pain.

[48] Delisle VC, Beck AT, Dobson KS, Dozois DJ, Thombs BD (2012). Revisiting gender differences in somatic symptoms of depression: much ado about nothing?. PLoS ONE.

[49] Kirmayer LJ, Robbins JM, Dworkind M, Yaffe MJ (1993). Somatization and the recognition of depression and anxiety in primary care. Am. J. Psychiatry.

[50] Bar KJ (2006). Decreased sensitivity to experimental pain in adjustment disorder. Eur. J. Pain.

