# Supplementary material for: Stress impacts sensory variability through cortical sensory activity motifs
Source: Transl Psychiatry. 2020 Jan 21;10:20. doi: 10.1038/s41398-020-0713-1 (PMC7026117; doi:10.1038/s41398-020-0713-1)
Supplement: Supplementary file 2 — Supplemental Figure legends [file 41398_2020_713_MOESM2_ESM.docx]

**Supplemental Figure Legends**

**Figure S1. Detection of sensory motifs in spontaneous VSD cortical activity at different thresholds using sensory-evoked templates.**

This montage illustrates ‘matches’ of the most distinctive frame of the 5 frame template and the frame within spontaneous activity for which the correlation coefficient crosses the threshold, represented at increments of 0.05SD of the mean correlation coefficient. Alongside the template and below each ‘match’ is the fluorescence signal dichotomized into presence/absence in order to compute the concordance between the template and the spontaneous ‘match’. The proportion of concordant dichotomized pixels between template and spontaneous ‘match’ is graphically illustrated at each threshold (*n*=8 animals). Forelimb template and matches are presented in **a&b)**, hindilmb template and matches are presented in **b&c)**, and whisker template and matches are presented in **d&e)**. We have not applied any spatial smoothing in these images as we wished to capture concordance with the template.

**Figure S2. Montages of null-hypothesis templates and data.**

**a)** Montage of the experimentally acquired templates by delivering standard stimulation protocols to the forelimb, hindlimb, and C2 whisker. Alongside these templates are examples of ‘shuffled’ templates generated from the experimental template. **b)** We generated one thousand animal specific ‘shuffled’ templates for each modality. Motif matches in experimental data fall outside of the 99% confidence interval represented by ‘shuffled’ template matches illustrated for ***i)*** a stressed animal and ***ii)*** a control animal. **c)** The population correlation distributions for ‘shuffled’ templates had an overrepresentation of zero and near-zero values relative to experimental templates (*n*=5 animals, 249,925 experimental template data points vs 249,925,000 ‘shuffled’ template data points) for forelimb (K-S=0.123, *p*<2.22*10^-308^), hindlimb (K-S=0.051, *p*<2.22*10^-308^), and whisker (K-S=0.0298, *p*=9.08*10^-193^). **d)** A montage of spontaneous cortical activity is presented alongside ‘shuffled’ activity generated from the same recording. **e)** We generated ten shuffled epochs of spontaneous activity (10,001 frames) for each animal. Motif match frequency in experimental spontaneous data far exceeds the match frequency identified in ‘shuffled’ spontaneous data for ***i)*** a stressed animal and ***ii)*** a control animal. When performed for 11 recordings from 5 animals, the population correlation distributions (109,967 experimental data points vs 1,099,760 ‘shuffled’ data points) revealed narrower distributions for **f)** forelimb (KS=0.124, *p*<2.22*10^-308^), **g)** hindlimb (K-S=0.155, *p*<2.22*10^-308^), and **h)** whisker sensory templates (K-S=0.157, *p*<2.22*10^-308^).

**Figure S3. Stressed animals have altered spontaneous cortical dynamics.**

**a)** Relative to controls, social defeated animals had broader correlation distributions (389,878 data points *n*=8 controls vs 449,865 data points *n*=9 defeated animals) for ***i*&*ii*)** forelimb (K-S=0.0727, *p*<2.22*10^-308^), ***iii*&i*v*)** hindlimb (K-S=0.0460, *p*<2.22*10^-308^), and ***v*&*vi*)** whisker stereotyped activity (K-S=0.0840, *p*<2.22*10^-308^). **b)** Maternally deprived animals had broader correlation distributions (689,793 data points *n*=14 facility reared vs 849,745 data points *n*=16 maternally deprived animals) for ***i*&*ii*)** forelimb (KS=0.0327, *p*<2.22*10^-308^), ***iii*&i*v*)** hindlimb (K-S=0.0476, *p*<2.22*10^-308^), and ***v*&*vi*)** whisker stereotyped activity (K-S=0.0123, *p*=2.23*10^-50^). **c)** ACC stimulated animals had broader correlation distributions (299,905 data points *n*=6 sensory cortex vs 349,895 data points *n*=7 ACC stimulated animals) for ***i*&*ii*)** forelimb (K-S=0.0460, *p*=5.50*10^-297^), ***iii*&i*v*)** hindlimb (K-S=0.0499, *p*<2.22*10^-308^), and ***v*&*vi*)** whisker stereotyped activity (K-S=0.0488, *p*<2.22*10^-308^). Error bars in graphs represent mean ± standard error.

**Figure S4. Sex-specific relationship between dominant motifs and active avoidance.**

a) The frequency of dominant motif occurrence was related to the degree of active coping on the FST **a)** in male animals (*F*(1,74)=8.95, *p*=0.0038), **b)** but not female animals (*F*(1,14)=0.01, *ns*).

**Figure S5. Weak cross-talk between ChrimsonR and iGluSnFR.**

Expression of Syn-ChrimsonR resulted in weak iGluSnFR cross-talk as demonstrated through decreased response amplitude in ChrimsonR expressing and non-expressing hemispheres (*t*(6)=2.76, *p*<0.05). Note that not only is the amplitude of the primary response decreased, there is an absence of a callosal response in the ChrimsonR expressing hemisphere.
